# Breaking Barriers—The Promise and Challenges of Limb Osseointegration Surgery

**DOI:** 10.3390/medicina61030542

**Published:** 2025-03-20

**Authors:** Agnieszka Wnuk-Scardaccione, Jan Bilski

**Affiliations:** Department of Biomechanics and Kinesiology, Institute of Physiotherapy, Chair of Biomedical Sciences, Faculty of Health Sciences, Jagiellonian University Medical College, 8 Skawińska Street, 31-066 Krakow, Poland; agnieszka90.wnuk@uj.edu.pl

**Keywords:** limb osseointegration, amputation, prosthetics, implants

## Abstract

Limb amputation remains a significant global health issue, affecting millions of individuals annually. A substantial proportion of these patients struggle with the inadequate fit and discomfort of conventional prosthetic sockets, leading to diminished quality of life. Osseointegration surgery, a promising alternative, offers the direct skeletal attachment of bone, implant, and prosthetic, providing a more stable and functional interface. Osseointegration remains an emerging procedure, and while exact global figures are difficult to pinpoint, estimates suggest that over 10,000 patients worldwide have received osseointegration implants since the technique was first introduced. This perspective article analyzes recent advancements in the field of limb osseointegration, highlighting key achievements such as improved implant materials, surgical techniques, and comprehensive prosthetic integration strategies. Additionally, it explores future directions for development and discusses the latest research trends shaping the evolution of this field. Despite these developments, the widespread adoption of osseointegration faces significant barriers, including complications, limited access to multidisciplinary care, economic constraints, and the need for further long-term clinical evidence. In this paper, we present an extensive perspective on the current state of osseointegration, discuss the challenges impeding its broader implementation, and offer recommendations to address these obstacles, with the goal of enhancing patient outcomes and facilitating the integration of osseointegration into mainstream clinical practice.

## 1. Introduction

Limb amputation represents a profound and transformative event, significantly impacting an individual’s mobility, quality of life, and daily functioning. In developed nations, lower-limb amputations are predominantly attributed to atherosclerosis and diabetes [1], while in less-developed regions, they are more frequently the result of traumatic injuries, often related to industrial accidents, traffic incidents, or combat-related trauma [2]. In recent decades, significant advancements in prosthetic technology have led to improvements in the design and functionality of artificial limbs. However, their utilization remains constrained by various limitations. Traditionally, the attachment of a prosthetic limb to the residual limb is facilitated through a custom-designed socket, which must ensure a secure fit to enhance comfort, effectively transfer the mechanical loads from the body to the ground through the intervening soft tissues, and enable the residual limb to control the prosthetic device [3]. However, several studies have reported dissatisfaction among prosthesis users, with approximately one-quarter of seventy-four participants and one-third of nine-hundred and thirty-five participants expressing dissatisfaction due to issues such as skin irritation, wounds, pain, and perceived poor quality of life [4,5]. These challenges have prompted the exploration and development of novel methods for the attachment of prosthetic components.

Osseointegration (OI) refers to the direct bonding of bone to a metallic implant, allowing for the attachment of an external prosthesis that passes through the skin and interfaces with the appendicular skeleton. This phenomenon was first identified through observations in animal models, where bone was seen to directly integrate with titanium implants, without the presence of intervening connective tissue [6].

In this context, the procedure of OI has emerged as a promising breakthrough in the field of prosthetics for patients following limb amputation. Issues identified in the literature, such as proper fitting of the prosthetic socket, frequently occurring stump volume changes, skin abrasions, neuropathic pain, and excessive perspiration [7], were expected to be effectively addressed through this approach. Furthermore, the direct integration of the prosthesis with the bone enhances proprioception, thereby reducing the energy expenditure associated with movement, lowering the risk of falls, and significantly improving the overall quality of life for patients following amputation [8,9].

Despite several decades having elapsed since the introduction of this revolutionary method and improvements in surgical techniques, the number of patients undergoing OI surgery remains relatively low. There is a lack of nationwide statistics comparing the outcomes of procedures performed in different countries. Additionally, the scientific literature does not provide a clear explanation for the potential reasons behind this phenomenon [10].

The aim of this article is to provide a comprehensive attempt to explain the potential logistical, administrative, and technical challenges and barriers, as well as the obstacles, associated with the widespread adoption of the OI surgery method.

## 2. Heterogeneity in the Limb Amputation Population

The greatest challenge for clinicians planning the treatment of individuals following amputation lies in the considerable heterogeneity within this patient population. The most significant differentiating factor is the primary cause of lower limb amputation, which, in European countries, is predominantly related to complications arising from diabetes management, peripheral arterial disease, infections (e.g., sepsis), as well as the sequelae of accidents and malignancies [11,12,13,14]. Amputations due to cancer, specifically as a result of tumors or malignant growths affecting bones or soft tissues, represent a smaller proportion of the overall number of amputations worldwide. However, they remain significant, particularly for patients with certain types of cancers, such as osteosarcoma and soft tissue sarcomas. Recent studies have indicated that amputations due to cancer are decreasing, thanks to advanced surgical techniques and early detection [15]. In contrast, in Africa, the leading cause is trauma resulting from road traffic accidents or armed conflicts [16]. The second most common etiology is infections secondary to poorly managed open wounds and malignancies [17,18,19]. In Asia, there is significant variability in access to medical information depending on the country. Japan and South Korea demonstrate notably lower rates of limb amputation, irrespective of the underlying cause. In low-income countries with higher population densities and significant industrial activity, the predominant causes are accidents and infection-related complications [20,21,22,23]. In the United States, the primary causes of amputations are chronic ischemia (72%), trauma (45%), and infection (15%) [24]. In South America, the primary causes of amputation align with global trends, with some regional variations [25]. In Australia, approximately 8,000 lower limb amputations are performed annually. Diabetes is a leading cause, and peripheral vascular disease, which involves the narrowing of arteries leading to the legs and feet, is another significant cause [26]. Epidemiological data on the distribution of the main causes of amputations worldwide are presented in Table 1.

In relation to the specific methodology of an OI procedure, where the implant is exposed externally, establishing a continuous connection between the internal and external environments, it is recommended that patients do not present an increased risk of wound healing complications or prolonged bone healing times. Some of the systemic conditions outlined above (e.g., diabetes mellitus or peripheral vascular disease) may exclude a patient from being considered a suitable candidate [27]. However, recent studies have reported good implant tolerance and favorable treatment outcomes even in individuals with higher risk profiles [28]. Nonetheless, there is still no definitive consensus on this matter. The preferred age of a patient undergoing the procedure remains a subject of debate. Most European and Australian studies recommend an optimal age range of between 18 and 70 years [29]. In the case of the U.S. population, the U.S. Food and Drug Administration (FDA) approves the procedure only for patients between the ages of 22 and 65 [30]. Additionally, the level of amputation and the length of the residual limb may pose barriers for potential candidates. In the case of below-knee amputations, the medical literature provides the most evidence supporting the potential benefits of the procedure [31]. For above-knee amputations, however, an adequate length of the residual bone is required or an additional endoprosthesis may be necessary to reinforce the remaining bone [32].

## 3. Varied Implant Technologies and Clinician Access

The technique of osseointegration (OI) has progressively gained broader acceptance over nearly three decades since its initial surgical implementation in Sweden on May 15, 1990. On this occasion, a patient who had previously undergone bilateral traumatic transfemoral amputations a decade earlier received the first stage of a titanium implant, which was anchored to one of their femurs. This pioneering approach was based on the foundational work of Per-Ingvar Brånemark, who, in 1965, discovered that titanium could form a robust and inseparable bond with rabbit bone [33]. The development of this technology led to the creation of the oldest and most extensively studied implant system used in OI procedures, the Osseointegrated Prosthesis for the Rehabilitation of Amputees (OPRA) [34,35]. The OPRA method consists of two separate surgical interventions for each bone, usually occurring 6 months apart. The initial procedure is centered on the placement of a threaded intramedullary bone fixation device. This process involves reaming the bone canal to the appropriate diameter, followed by tapping the threads to prepare the site for the implant, which is then screwed into place, ensuring it is inserted to a depth of at least 20 mm beyond the distal bone edge. This depth is critical in preventing potential bone resorption. Once the implant is secured, the incision is fully closed. During the healing period, the extremity is kept non-weight-bearing or the patient may use a conventional socket prosthesis to avoid loading the bone while the osseointegration process is in its early stages. After 6 months, allowing sufficient time for the implant to integrate with the host bone, the second surgical procedure is performed [36]. Another current possibility is The Integral Leg Prosthesis (ILP) implant that was derived from the Endo-Exo implant (ESKA Orthopaedic Handels), a system initially introduced by Hans Grundei in Germany. This system was also developed for two-stage implantation. The ILP system offers greater flexibility through a modular design that emphasizes soft tissue integration [37]. The Osseointegrated Prosthetic Limb (OPL), developed by Al Muderis, was designed for single-stage surgery implantation, representing the first implant created specifically for this purpose. In patients with extremely short residual limbs (less than 8 cm), bone lengthening can be accomplished using an externally activated intramedullary magnetic expandable nail. Once the desired length is attained and the bone has fully consolidated, the standard osseointegration procedure is then initiated [38]. Since the introduction of the single-stage protocol in 2014, nearly all patients have undergone only one surgical procedure rather than the traditional two-stage approach, followed by a standardized rehabilitation regimen [39]. The Compress system, originally developed for oncological limb salvage surgeries, is an endo-prosthetic device that is inserted into the residual bone via a press-fit mechanism. The intramedullary stem of the system is fixed to the bone with the use of transverse pins. A spindle positioned at the distal end of the residual bone generates a compressive force, which promotes bone growth. This force is designed to prevent the implant from loosening in an aseptic manner [40]. Table 2 presents a global comparison of three implant systems in limb osseointegration.

The choice of the appropriate method and implant type largely depends on the surgeon managing the patient post-amputation, with the final decision often being influenced by the patient’s geographical location and the standard techniques commonly used in that region. In the United States, the only FDA-approved method for osseointegration is the OPRA system. In European countries (primarily Germany, Sweden, and the Netherlands), OPRA is the preferred method, although other surgical techniques are also permitted. In Australia, the most widely adopted method is the OPL system, primarily due to the extensive research conducted under the leadership of Professor Munjed Al Muderis [41,42]. While exact data on the number of specialists performing these procedures are lacking, it is estimated that the number does not exceed 100 specialists globally, given the requirement for specialized teams, equipment, and rehabilitation infrastructure. Furthermore, only a few European countries fully cover the cost of the procedure (Sweden, Germany, The Netherlands, France, the UK, Denmark, and Finland), with a majority of the surgeries being funded privately by patients. Some countries, like Sweden and Germany, may require prior approval or specific documentation from healthcare providers to qualify for full insurance coverage. The cost of limb osseointegration varies substantially depending on the country, hospital, and specific implant system used. On average, patients can expect to pay anywhere from USD 15,000 to 100,000 globally. It is important to note that there is no official, standardized information that establishes the cost of osseointegration surgery worldwide [43]. For many potential candidates, the cost of the procedure remains prohibitive.

## 4. Potential Risks and Complications

OI surgery has evolved since its inception, leading to a reduction in the associated risks and complications. However, it has not been able to completely eliminate these risks and complications. One of the most frequently cited complications in the literature remains the risk of infection, which accounts for approximately 32% of all complications [44,45]. Given that the distal end of an implant maintains a continuous and direct connection with the external environment, distinguishing between a normal skin reaction due to irritation and a bacterial infection is not a straightforward task. The types of infections examined include superficial or stoma infections, deep tissue infections such as osteomyelitis, and peri-implant infections. To confirm the presence of an infection, a bacterial culture test should be performed [46]. Staphylococcus, a genus of Gram-positive, facultative anaerobic bacteria, is responsible for up to two-thirds of the pathogens identified in orthopedic implant infections [46]. While previous studies have demonstrated that infections are relatively common following lower-limb orthopedic implantations, there is a lack of comprehensive evidence regarding the etiology, progression, and management of these infections in the context of OI. The primary challenge remains that most patients with infections seek care from general practitioners in their local area, who are easily accessible to them. Given that osseointegration is a relatively uncommon procedure, many physicians encounter such cases infrequently in their practice, resulting in treatment approaches that may be inadequate for the specific needs of these patients [47]. Recent Australian studies have indicated that peri-implant pain, particularly when accompanied by apparent local cellulitis, is strongly indicative of infection, whereas inflammatory markers appear to have limited diagnostic value [48].

Fractures linked to OI have the second-highest occurrence, impacting 3.31% of patients. In comparison to conventional amputations with socket-suspended prostheses, this could represent a potential disadvantage of OI use. The existing literature indicate that patients with socket-suspended prosthetics also face a 2.2% fracture risk over a five-year period [49,50]. In most cases, data regarding the potential for fractures are based on relatively short follow-up periods. The longest reported follow-up was 10 years and pertained to a specific cohort of patients who underwent amputation due to cancer. Fractures were observed in only 3 out of 17 patients during this period, representing a small proportion of the total cohort [51]. An Australian center conducted an assessment of osseointegration outcomes over 8 years, involving 518 procedures, reporting a total of 22 fractures [52]. Considering the available literature, the risk of fracture should not deter clinicians or potential candidates from undergoing this surgical procedure.

A potential complication worth noting is the loosening of the implant or the need for its removal. The incidence of such events among patients following osseointegration is estimated to be approximately 2.98% [53]. Similarly, in the group of amputee patients using a prosthetic socket, revision procedures are required in approximately 25% of all cases [54].

**Figure 1:**
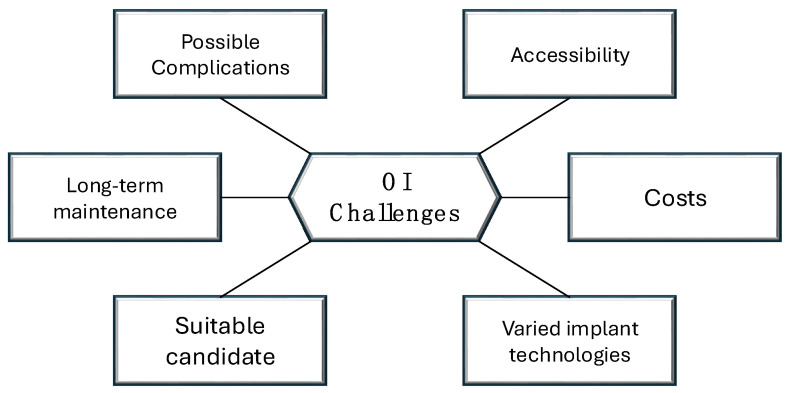
Key challenges facing the development of osseointegration.

According to a recent literature review summarizing all known complications following limb osseointegration procedures, other undesirable events such as soft tissue neuroma formation, postoperative systemic inflammatory response syndrome (SIRS), decreased activity and mobility, soft tissue arthrofibrosis, metastatic disease, local disease recurrence, soft tissue impingement, chronic pain, and even death are reported to occur very rarely, with no substantial risk to patients [55]. Despite this, ongoing advancements in implant materials and design, particularly regarding sizing and biomaterials, continue to focus on minimizing the occurrence of these complications over time. The main challenges associated with osseointegration surgery are presented in Figure 1.

## 5. Patient Selection for Limb Osseointegration: Key Considerations

At present, the indications for lower-extremity osseointegration are relatively limited, with the procedure primarily serving as a secondary option for individuals who cannot tolerate socket-based prostheses. It is important to emphasize that osseointegration is not a life-saving procedure, but rather, it is aimed at improving quality of life. Therefore, the time allocated for patient education, systemic preparation, and rehabilitation prior to surgery should be sufficiently long [56].

The largest group of patients who have successfully undergone osseointegration procedures consisted of veterans and individuals who had undergone amputations due to traumatic causes [57]. Most of these patients were young, relatively healthy individuals with good muscular strength, superior bone quality, and objectively better conditions for healing. Nevertheless, there was also a large group of patients who reported persistent pain in their lower backs, which significantly reduced their quality of life [58]. While age does influence the decision-making process, particularly in terms of bone quality and healing potential, there is no fixed age limit for limb osseointegration.

In high-income countries, the most significant challenge lies in managing diabetic patients, as this group is particularly prone to lower-extremity amputation. Following prosthetic fitting, these patients often experience skin-related issues beneath traditional prosthetic sockets [59,60]. Vascular pathology, a common comorbidity in this population, remains one of the primary contraindications for osseointegration. The key reason for this contraindication is the lack of sufficient bone quality for implant placement. A healthy bone bed is essential for initiating the de novo bone deposition process at the bone–implant interface, a process mediated by macrophages [61]. Consequently, patients with osteopenia or osteoporosis or those who have undergone chemotherapy or radiation therapy are typically excluded from osseointegration procedures. However, there are emerging reports suggesting that such patients should not be entirely excluded from consideration for the procedure. Small-scale studies have demonstrated the procedure’s efficacy and highlighted the significant functional benefits it can offer to these patients [27,62]. These data represent preliminary findings, but they indicate a potential direction for change, offering hope for the expansion of eligible candidates for the procedure.

One of the key factors in the success of all orthopedic procedures is a well-planned loading protocol for the operated limb, coupled with appropriate rehabilitation. In the case of OI, there is a lack of standardized protocol. Recommendations and consistent management depend on the type of implant used and the multidisciplinary team overseeing the patient’s care. While a majority of the OI-related literature focus on the surgical techniques, technology, basic science, or complications associated with OI, rehabilitation strategies and related challenges remain underrepresented [63]. An Australian team has proposed a week-by-week protocol for rehabilitative management [64], whereas for other types of implants, only general recommendations and guidelines can be found. When recruiting a candidate for OI surgery, it is essential to consider psychological factors. Patients must demonstrate psychological readiness, an understanding of the benefits and risks, and a willingness to engage in post-operative rehabilitation. Attention should also be given to patients who place excessive expectations on the procedure, such as expectations that do not align with the realistic benefits achievable. This is particularly important given that most potential candidates base their knowledge of the procedure entirely on information found online [65]. The biopsychosocial model should involve conducting appropriate psychological testing, with potential tests including the Body Image Scale (BIS), the Anxiety and Depression Scale (HADS), or the Patient Health Questionnaire (PHQ-9) [66]. In the case of assessing motor skills before and after the surgery, the Amputee Mobility Predictor (AMP) or Functional Independence Measure (FIM) could be utilized [67]. It would be advisable to introduce a standardized tool for assessing the functional and psychological readiness of a patient undergoing osseointegration surgery. This tool should include components such as pain assessment, previous prosthetic use, range of motion, and prior quality of life. The tests should be standardized and conducted prior to each osseointegration procedure, regardless of the location where the surgery is performed. It is worth noting that in countries where osseointegration is not reimbursed, the economic factor becomes a crucial consideration. The patient should be aware of the procedure’s costs, but this aspect must not outweigh the importance of meeting the appropriate medical and psychological criteria for candidacy [68]. The requirement for patients to independently finance the osseointegration procedure may significantly limit its accessibility, particularly in cases where the procedure is highly recommended due to a marked reduction in quality of life (e.g., difficulty in using a prosthetic socket). Conversely, it may also pose a risk for patients who can afford the procedure but it is not advisable due to additional medical conditions or contraindications.

## 6. Innovations and Future Directions

When considering the risks and complications associated with the OI procedure, a key direction for the future is improving the implant itself. All osseointegration (OI) implants, irrespective of their design or surgical technique, encounter similar challenges, with transdermal implants being particularly prone to infection. To address these concerns, current strategies involve modifications to the implants, including the application of antibiotic or silver nanoparticle coatings [69,70]. Although these methods have demonstrated encouraging results in pre-clinical studies, they have yet to be evaluated in human trials, and their actual effectiveness remains unclear.

An alternative method for preventing infection includes the application of external electrical stimuli to the titanium implant. A recent study investigated the effects of cathodic electrical stimulation on biofilm formation. In traditional implants, Gram-positive bacteria have the ability to create a glycocalyx biofilm, which acts as a barrier to antibiotic penetration, thereby enhancing their resistance to antibiotic treatment [71]. Only a limited number of studies involving different animal species, including rabbits, dogs, and sheep, have explored the use of electrical stimulation with direct coupling for the osseointegration of implants [72,73]. Considering these recent advancements, employing direct coupling of electrical stimulation via an alternating field could offer a promising therapeutic approach to enhance the osseointegration of permanent bone implants, particularly in patients with compromised bone metabolism. However, it is still in the pre-clinical stage, and clinical implementation would necessitate determining the appropriate dose–response relationship and optimal duration of stimulation, especially in patients with relevant diseases [74].

The OI procedure facilitates the more precise control of prosthetic movements by utilizing the concept of osseoproprioception [75]. By directly anchoring a prosthetic limb to bone, this approach offers greater natural movement and functionality in comparison to traditional socket-based systems. To address issues like painful neuromas and to improve the fidelity of long-term signaling, the Osseointegrated Neural Interface (ONI) technique has been developed [76]. This innovative technique combines principles of osseointegration with nerve regeneration, creating a direct interface between the peripheral nerves and an advanced prosthetic. The fundamental idea is that the intramedullary canal can create a protective environment that supports nerve healing and ongoing physiological function. Studies conducted using rabbit models have shown that nerves incorporated into bone tissue can transmit both motor (efferent) and sensory (afferent) action potentials, which are crucial for prosthetic control and for transmitting sensory information to the central nervous system. While the ONI technique has shown clinical efficacy in treating neuropathic pain [76,77] and significant evidence supports the use of osseointegration, the combined application of these technologies in human subjects remains an area that has yet to be fully explored through proper clinical testing [78].

**Figure 2:**
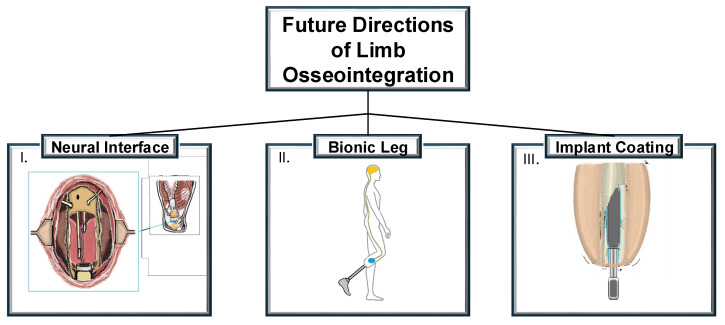
The future trends in osseointegration development are as follows: (I) A technique that merges osseointegration principles with nerve regeneration, establishing a direct connection between peripheral nerves and a sophisticated prosthetic. It is still in the preclinical stage, and ongoing research continues to explore its potential for future clinical applications. (II) The direct integration of robotic elements with an amputee’s body, where technology has progressed beyond preclinical trials and is undergoing clinical evaluation to assess its effectiveness and potential benefits for amputees. (III) The use of antibiotic and silver nanoparticle coatings on implants for limb osseointegration, which are primarily in the preclinical phase.

The integration of robotic components directly with the body of an amputee represents a promising direction for the future. Integrating bionic limbs with the human body involves addressing various challenges, including the attachment of prosthetics, the development of effective human–machine interfaces, the control of a prosthetic, and the rehabilitation and training required for users. The example of the Utah Bionic Leg, developed by the University of Utah, is undergoing clinical trials to evaluate its effectiveness in improving mobility for individuals with above-knee amputations [79]. Next-generation prosthetic approaches should integrate both reliable control systems and chronically implanted electrodes. Muscle interfaces are expected to be the primary focus for control, given the substantial limitations of neural- and brain-based interfacing. Implantable wireless muscle sensors, such as IMES (implantable myoelectric sensor), have already undergone clinical trials and will likely become more prevalent in limb prosthetics [80]. The iSens system, which integrates muscle electrodes implanted for control with neural stimulation to provide sensory feedback, demonstrates considerable potential, although it remains in the pre-clinical trial stage [81]. However, its high energy consumption and current incompatibility with existing osseointegration methods may limit its widespread application. Implantable electromyographic sensors, utilizing algorithms similar to those used in surface electromyography, will be employed to control a prosthesis, ensuring stable and high-fidelity signals. The integration of muscle bioscreens with implanted muscle sensors is expected to improve the stability of the interface and significantly enhance the functionality of the prosthesis–neural system connection [82]. As suggested by research, in the future, it may be possible to reduce unnecessary surgeries and simultaneously perform amputation and osseointegration [83]. The key trends in the future development of OI surgery are presented in Figure 2. To accelerate pre-clinical trials for technologies, several policy changes could be beneficial. Expanding insurance coverage for pre-clinical trials and offering partial reimbursement for participation could reduce financial barriers for both researchers and patients. Establishing global clinical trial registries and standardizing data reporting would improve collaboration and streamline regulatory processes. Public–private partnerships and tax incentives for research and development would encourage faster technological advancements. Additionally, fostering collaboration between academic institutions, hospitals, and private companies could accelerate trial recruitment and data gathering.

The future projections for limb osseointegration surgery are supported by a robust research pipeline that is focusing on implant coatings, neural interfaces, and improved surgical practices. These developments suggest that significant advancements in the field are not only likely but also imminent.

## 7. Conclusions

Osseointegration offers significant potential to support amputees who are unable to tolerate conventional prosthetic suspension methods. However, despite the promising advances in this field, the technology remains in its early stages and has yet to bring about a transformative change in the lives of patients following limb amputation. The key challenges include the lack of long-term, multi-center studies; the existence of competing implant systems and surgical approaches; and the absence of unified rehabilitation protocols. The economic aspect of osseointegration also presents two major concerns: first, the procedure is not funded by most healthcare systems, making it unaffordable for many patients; and second, when funding is available, it often results in high-risk patients being operated on, increasing the likelihood of complications and failures. The clinical issues associated with osseointegration, such as infections and loss of integration, require ongoing monitoring to prevent adverse outcomes.

### Future Perspectives

To foster progress in this field, a collaborative effort across multiple disciplines is crucial. The following actions can be taken to pave the way for broader clinical applications and improve patient outcomes:

- To build a strong evidence base, it is essential to fund and conduct large-scale, multi-center trials that track the long-term outcomes of osseointegration.
- Unified, evidence-based rehabilitation protocols should be developed to ensure consistency in post-surgical care and improve patient recovery outcomes. These guidelines should encompass both physical rehabilitation and psychological support, as adapting to the new prosthetic system can be a challenging process.
- Governments and healthcare systems need to recognize the long-term benefits of osseointegration and explore funding options to make this technology more accessible to patients. Public–private partnerships could be key in making osseointegration more affordable while also advancing research into lower-cost, equally effective implant options.
- The future of amputee rehabilitation will require the seamless integration of osseointegration with other technologies such as targeted muscle reinnervation, myoelectric sensors, and sensory feedback systems. A coordinated approach that combines these innovations could significantly improve the quality of life for an amputee, offering them greater independence and functionality.

## Figures and Tables

**Table 1:** The main causes of amputations, categorized by relevant regions of the world based on available epidemiological studies.

Region	Country	References	Time of Observation	Primary Causes	Secondary Causes
Europe	Ireland	Maely A./2022 [12]	2016–2019	Of the total number of all 3104 amputations, approximately 51.3% (*n* = 1592) of the minor amputations and 16.9% (*n* = 525) of the major amputations were performed on patients with diabetes diagnoses	-
Europe	Romania	Rusu E/2023 [13]	2015–2019	Of the total number of non-traumatic amputations, 51.2% were performed on patients with diabetes (*n* = 40,499)	Trauma-related, 11.4% (*n* = 9013)
Europe	England	Ahmad *n*/2014 [14]	2003–2009	There were 25,312 major lower limb amputations, and the most common disease risk factors were diabetes (43.7%) and hypertension and coronary heart disease (39%)	Trauma-related, 12%
Africa	Ethiopia	Sume BW/2023 [16]	2000–2022	The authors reviewed18,900 study participants, and the major cause of limb amputations was trauma (11.05%), with traditional bone setters (24.10%)	Burn, 10.63%; diabetic foot ulcer, 9.93%
Africa	Nigeria	Al-Ajlouni YA/2024 [18]	1991–2005	There were 1642 amputations, and the most frequent indications for amputation were trauma (34%), complications in traditional bone-setting (TBS) (23%), and malignant tumors (14.5%)	Diabetic gangrene, 12.3%; infections, 5.1%; peripheral artery disease, 2.1%; and burns, 2.1%
Asia	Bangladesh	Hassan Al Imam M/2020 [21]	2014–2016	Of the 332 participants, road traffic accidents were the leading cause (58.7%)	Peripheral vascular diseases, 7.5%; hit by sharp objects, 7.2%
Asia	South Korea	Chung HJ/2022 [23]	2011–2018	There were 8156 amputation cases, with the leading causes being peripheral vascular disease (PAD) (63.7%) and diabetes (32.1%)	-
North America	USA	Rivera JA/2024 [24]	2016–2019	There was a total of 2,118,175 amputations, with PAD and diabetes (83%) being the leading causes	Trauma-related, 9%
South America	Brazil	Portela FSO/2024 [25]	2008–2020	There were 633 455 amputations, with PAD (62.7%) and diabetes (23%) being the leading causes	Trauma-related, 11%
Australia	Australia	Dillon MP/2017 [26]	2007–2012	There was a total of 35,306 amputations, with diabetes (44.6%) and PAD (32.2%) being the leading causes	Trauma related (9.5%), Cancer (5.4%)

**Table 2:** Comparison of the advantages and disadvantages of three implant systems used in limb osseointegration (OPRA, Compress, and OPL).

Implant System	Advantages	Disadvantages
**OPRA**	Proven long-term success for both upper- and lower-limb amputees	Higher risk of infection due to concentrated stresses around the threads
	The design features enhance torsional stability and enable implantation in cases with a short residual limb	Possible complications with soft tissue overgrowth and irritation
	High patient satisfaction with functional outcomes	Expensive, with a long two-stage surgical process
**Compress**	Minimally invasive surgical procedure	Limited clinical data compared to OPRA; long-term outcomes still being studied
	Potential for a lower risk of infection due to internal components	Potential for less stability in certain cases, particularly with bone quality, higher risk of aseptic loosening
	Simplicity of performing revision surgery	Stress shielding or the removal of physiologic stress on bone by an implant may lead to osteopenia and reduced cortical thickness
**OPL**	Designed to minimize the risk of infection as the polished adapter helps prevent fractures in the surrounding bone	Limited long-term clinical evidence
	One-stage procedure, faster loading of the operated limb	The complexity of internal design could potentially lead to greater difficulty in adjustments, repairs, or revisions
